# Genetic Analysis of Tropical Midaltitude- Adapted Maize Populations under Stress and Nonstress Conditions

**DOI:** 10.2135/cropsci2017.09.0531

**Published:** 2018-06-07

**Authors:** Dan Makumbi, Silvano Assanga, Alpha Diallo, Cosmos Magorokosho, Godfrey Asea, Mosisa Worku, Marianne Bänziger

**Affiliations:** 1 International Maize and Wheat Improvement Center (CIMMYT), PO Box 1041-00621, Nairobi, Kenya;; 2 Monsanto Company, 1506 Hwy 69 Suite 100, Waco, NE 68460, USA;; 3 Guinee-Semences, Immeuble Guinomar, Camayenne, Corniche Nord, BP 5603, Conakry, Guinea; 4 International Maize and Wheat Improvement Center (CIMMYT), PO Box MP 163, Harare, Zimbabwe;; 5 National Agricultural Research Organization, National Crops Resources Research Institute, Namulonge, PO Box 7084, Kampala, Uganda;; 6 International Maize and Wheat Improvement Center (CIMMYT), Apdo

## Abstract

Maize (*Zea mays* L.) yield in sub-Saharan Africa (SSA) is low because of both abiotic and biotic constraints, and limited availability or use of improved seed in some areas. This study was conducted (i) to estimate combining ability and heterosis among seven stress-tolerant populations, and (ii) to assess diversity among the populations and the relationship between diversity and heterosis. Twenty-one hybrids developed from diallel crosses of seven populations, parents, and two checks were evaluated in 10 optimal and 11 stressed environments (drought, low N, and random stress) in Kenya, Ethiopia, Uganda, and Zimbabwe for 2 yr. Analysis II of Gardner and Eberhart showed that variety and heterosis were significant for grain yield (GY) under optimal and managed stress, and across environments. Heterosis accounted for most of the variation for GY among populations under optimal conditions (67%) and drought stress (53%), which suggested the importance of dominance in inheritance of GY under these conditions. Genetic distance (GD) among populations ranged from 0.328 to 0.477 (mean = 0.404). The correlation between GD and heterosis was low (*r* = 0.14–0.40) in all environments. The simple sequence repeat (SSR) marker-based and GY-based clustering of parental populations showed similar patterns, with three populations distinct from the rest, suggesting significant differentiation of allelic variation in these three populations. The SSR-based diversity and phenotypic analysis results should be useful in defining breeding strategies and maintaining heterotic patterns among these populations.

Maize (*Zea mays* L.) is one of the leading cereal crops worldwide in grain production and ranks second after rice (*Oryza sativa* L.) in economic value (FAOSTAT, 2014). Total maize production in sub-Saharan Africa (SSA) was estimated to be 65 Tg in 2014, of which 45 Tg were produced in eastern and southern Africa (ESA) (FAOSTAT, 2017). Predictions are that the demand for maize for food and feed across developing countries will overtake that for wheat (*Triticum aestivum* L.) and rice by 2020 (IFPRI, 2000). By 2025, maize is expected to be the crop with the highest production globally (Rosegrant et al., 2008). Maize is a major staple food crop in ESA, where it is an important source of carbohydrates and proteins, accounting for 17 to 60% of people’s total daily protein supply (Krivanek et al., 2007).

Maize yield in SSA averages 2.1 Mg ha^–1^, which compares poorly with the world average of 5.6 Mg ha^–1^ (FAOSTAT, 2017). Most farmers in the SSA region grow maize under rainfed conditions and face a multitude of production challenges. Among the most important production constraints are abiotic stresses (drought, heat, and low soil fertility), biotic stresses (mainly *Maize streak virus* [MSV], maize lethal necrosis, foliar diseases, and insect pests), and limited availability of improved seed in some areas. Working with national maize breeding programs, CIMMYT and IITA have addressed many of these constraints through the development and dissemination of stress-tolerant maize varieties in SSA (Bänziger et al., 2006; Badu-Apraku et al., 2013; Setimela et al., 2017).

In ESA, farmers plant a combination of improved maize hybrids, open-pollinated varieties (OPVs), their own saved maize grain (recycled seed), landraces, and local cultivars. Hassan et al. (2001) estimated that hybrids occupied ~46% of the maize area planted in SSA, whereas only 7% was planted with improved OPVs. A recent study has indicated that ~32% of maize varieties grown in SSA are hybrids, 23% are improved OPVs, and the rest are landraces and local cultivars (Abate et al., 2017). Hybrids reportedly contributed 78% of total 103,600 metric tons of improved maize seed supplied in 2006 and 2007 in ESA (Langyintuo et al., 2010). Improved OPVs are important to those farmers in SSA who do not readily buy hybrid seed every year because of a number of constraints. In some farming systems under smallholder conditions, recycling improved OPVs may be more profitable and sustainable than purchasing fresh hybrid seed (Pixley and Bänziger, 2004; Pixley, 2006). Currently, improved OPVs are developed by intermating elite inbred lines of similar maturities or recombining full-sib families (Pixley et al., 2006). Open-pollinated varieties that combine high grain yield (GY), tolerance to abiotic stresses, and resistance to biotic stresses have been developed and commercialized in ESA (Pixley and Bänziger, 2004; Setimela et al., 2007; Vivek et al., 2009; Masuka et al., 2017). Improvement in performance of stress-tolerant OPVs under different conditions across years has been reported; for example, Masuka et al. (2017) found that genetic gain in early-maturing OPVs under optimal conditions, random drought, and low N was 109.9, 29.2, and 84.8 kg ha^–1^ yr^–1^, respectively, whereas for intermediate- to late-maturing OPVs, genetic gain under similar conditions was 79.1, 42.3, and 53.0 kg ha^–1^ yr^–1^, respectively.

The CIMMYT maize breeding programs in Kenya and Zimbabwe use germplasm from different sources to develop diverse improved stress-tolerant OPVs of different heterotic patterns (A and B). Open-pollinated varieties are formed for direct use as varieties, whereas narrow-based synthetics (involving 8–10 inbred parents) are used as source populations for inbred line development. Diversity in such broad- or narrow-based populations can be analyzed through phenotypic evaluation of morphological traits in multiple environments, by using molecular markers, or by a combination of both approaches. Molecular markers have been used to investigate diversity among OPVs and pools in CIMMYT’s maize germplasm (Warburton et al., 2002, 2008; Semagn et al., 2014) and in maize germplasm from other regions (Rebourg et al., 2001; Gauthier et al., 2002; Reif et al., 2005; Noldín et al., 2016). The use of simple sequence repeat (SSR) markers is effective in assessing genetic diversity and grouping of broad-based populations (Warburton et al., 2002; Reif et al., 2003b; Semagn et al., 2014). Diversity analysis of maize populations is helpful in developing strategies to use genetic resources in breeding improved stress-tolerant populations, OPVs, and synthetics. Several populations have been developed by CIMMYT using a large number of inbred lines from diverse sources, and it is important to understand the genetic relationships among these populations for their better utilization in breeding programs. The objectives of this study were (i) to estimate combining ability and heterosis among seven stress-tolerant populations of diverse genetic backgrounds, and (ii) to assess diversity among the populations and to estimate SSR molecular marker-based genetic distance (GD) and the relationship between diversity and heterosis.

## MATERIALS AND METHODS

### Genetic Materials

Seven maize populations (two broad-based populations, one OPV, and four synthetics), developed between 1997 and 2000 at CIMMYT in Kenya, were used in this study. The inbred lines used to form the populations were selected on the basis of good combining ability for GY and resistance to the major foliar diseases, such as northern corn leaf blight [NCLB, caused by *Exserohilum turcicum* (Pass.) K. J. Leonard & Suggs], gray leaf spot (GLS, caused by *Cercospora zeae-maydis* Tehon & E. Y. Daniels) and MSV, which are economically important in the midaltitude ecology of ESA. In addition, some of the parental lines were also selected on the basis of their tolerance to other biotic (insects) and abiotic (drought and low N) stresses. The populations were coded “ECAVL” (eastern and central Africa variety of late maturity) or “NIP” (non-inbred parent). Further descriptions of these populations can be found in the paragraphs below.

ECAVL1 is a population formed by intermating 82 inbred lines, including eight lines adapted to the midaltitude region of ESA and 74 inbred lines extracted from four Mexican lowland tropical- and subtropical-adapted populations (43SR, 501, THG-A, and 590). Population 43SR is a late-maturing, lowland tropical-adapted population of Tuxpeño type, improved for resistance to MSV. Population 501 is a subtropical population of intermediate maturity and contains temperate germplasm from the United States. Population THG-A is a Tuxpeño-type tropical population of late maturity composed of lines extracted from Populations 21, 22, 29, and 43 and Pool 24 (CIMMYT, 1998). Population 590 is a tropical-adapted population of late maturity and is known as the multiple borer-resistant population. The eight lines adapted to ESA were extracted from population EV7992#/EVPOP43BC3-SR3.

ECAVL2 is a population formed by intermating 78 inbred lines, including 15 lines adapted to the midaltitude region of ESA and 63 inbred lines extracted from four Mexican tropical- and subtropical-adapted populations (22SR, 502, THG-B, and 590). Population 22SR is a late-maturing tropical population of Tuxpeño and ETO Blanco background, improved for resistance to MSV. Population 502 is a subtropical population of intermediate maturity and contains temperate germplasm from the United States. Population THG-B is a Tuxpeño-type tropical population of late maturity, composed of lines extracted from Populations 22, 25, 29, 32, 43, and 73, Pools 23 and 24, and Tuxpeño Sequía. The 15 lines adapted to ESA were: CML201, CML202, CML204, and CML212; eight lines extracted from population M37W/100MSR; and three lines extracted from population MSR131.

ECAVL16 is an OPV formed by intermating 21 inbred lines, of which seven lines were adapted to the midaltitude region of ESA and 14 lines were from six Mexican tropical- and subtropical-adapted populations (P43C9, 390, 500, Tuxpeño Sequía6 C2, La Posta Sequía C3 and C7, and Pool Phyllacora C0). Population 43 is a late-maturing, lowland tropical-adapted population of Tuxpeño type. La Posta Sequía C3 and C7, and Tuxpeño Sequía6 C2 are populations improved for drought tolerance (Edmeades et al., 1999). Population 390 is of the late maturity category and represents multiple insect-resistant or -tolerant germplasm. Population 500 is a subtropical intermediate-maturing population derived from temperate, Asian, and subtropical germplasm. Pool Phyllacora C0 is a population with resistance to *Phyllachora maydis* Maubl., which is one of the causal agents of the tar spot complex. The seven lines adapted to ESA were extracted from populations MSRXPOOL9, AC8342, FR810/TZMSR, and EV7992#/EV8449SR.

ECAVL16STR is a synthetic formed by intermating 13 inbred lines extracted from six Mexican tropical-adapted populations (P43C9, La Posta Sequía C3, La Posta Sequía C7, P22SR, Tuxpeño Sequía6 C2, and Pool Phyllacora C0). The lines used to form this synthetic showed tolerance to *Striga hermonthica* (Del.) Benth. in testcross trials conducted in Kenya.

ECAVL17 is a narrow-based synthetic formed by intermating eight inbred lines, of which three were adapted to ESA and five were adapted to tropical lowland Mexico. The component lines were CML78, CML202, CML312, CML373, CML379, CML442, and one line each from populations La Posta Sequía C3 and EV792 × EV8449-SR. Inbred lines CML202, CML312, and CML442 are among the most widely used parents in commercial hybrids in ESA.

ECAVL18 is a narrow-based synthetic formed by intermating eight inbred lines—five adapted to ESA and three adapted to tropical lowland Mexico. The component lines were CML216, CML247, CML384, CML395, CML441, and CML444, and one line each from Tuxpeño Sequía C1 × P49-SR and AC8342 × 8149-SR. Inbred lines CML395 and CML444 are among the most widely used parents in commercial hybrids in ESA.

NIP25 is a narrow-based synthetic formed by intermating eight inbred lines extracted from Population 25. Population 25 is a tropical lowland population of intermediate to late maturity from the Blanco Cristalino-3 background. More details about the breeding procedure and composition of all the populations from Mexico, from which the lines were extracted, are provided in CIMMYT (1998; http://repository.cimmyt.org/xmlui/handle/10883/757). The synthetics developed were maintained as OPVs.

The seven populations were crossed using a diallel mating design in 2007. Crosses were made at the Kenya Agricultural and Livestock Research Organization (KALRO) Kiboko Research Center, Kenya. Each population was planted in a nursery block of 105 plants. Bulk pollen was collected from 20 to 30 plants from each entry and used to pollinate receptive silks of 20 to 30 plants in the corresponding entry. This was repeated until the majority of the plants in each entry had been pollinated. To capture genetic variation within the populations, we broke the tassel of a plant after collecting its pollen to ensure that no plant was used as a pollen source more than once. Each entry was used as both male and female. Seed of the reciprocal crosses was bulked to form 21 population hybrids.

### Test Locations, Experimental Design, and Trial Management

The 21 population hybrids, their seven parents, a commercial check hybrid (WH403) common across all trials, and a local check hybrid were grown in 21 trials planted at six locations in Kenya and one location each in Ethiopia, Uganda, and Zimbabwe in 2008 and 2009 (Table 1). The experimental design was a five-by-six α-lattice (Patterson and Williams, 1976) with three replications. Each experimental unit consisted of two rows spaced 0.75 m apart and 0.25 m between plants, giving a population density of ~53,333 plants ha^–1^ at all locations, except Kiboko, where the spacing was 0.75 m between rows and 0.20 m between hills to give a final plant density of ~66,666 plants ha^–1^. Standard agronomic and cultural practices were performed as recommended for each location. The trials were planted as follows: 10 trials (five locations × 2 yr) under optimal conditions, four trials (two locations × 2 yr) under low N, four trials (two locations × 2 yr) under random abiotic stress, and three trials under managed drought stress (one location × 2 yr, and one location × 1 yr) (Table 1). Trials planted under optimal management were entirely rainfed. In 2009, the rainfall distribution was erratic at some locations, resulting in reduced yield because of random drought. Therefore, the categorization of trials under rainfed conditions into optimal and random abiotic stress was slightly different than that suggested by Weber et al. (2012). A trial was considered to be under random abiotic stress if mean GY was <3.3 Mg ha^–1^ in both years. A trial was considered to be under optimal conditions if the mean GY of the trials was >3.3 Mg ha^–1^ across the 2 yr.

**Table 1:** Test locations characteristics (coordinates, management, rainfall, and temperature), fertilizer application rates, and trial mean grain yield in 2008 and 2009.

Location	Country	Latitude	Longitude	Elevation	Management	Fertilizer application rate	Rainfall		Temperature (min, max)		Mean grain yield	
							2008	2009	2008	2009	2008	2009
				m		kg ha^−1^	—— mm ——		—— °C ——		— Mg ha^−1^ —	
Bumula	Kenya	0°63′ N	34°51′ E	1383	Random abiotic stress	37 P, 97 N	682	560	17.3, 25.6	17.4, 27.1	3.23	2.93
Busia	Kenya	0°30′ N	34°18′ E	1250	Random abiotic stress	37 P, 97 N	1248	1034	19.6, 24.9	19.9, 25.7	0.96	1.72
Elgon Downs	Kenya	1°05′ N	34°51′ E	1876	Optimal	37 P, 97 N	746	438	12.9, 24.7	12.5, 25.9	5.54	2.67
Embu	Kenya	0°30′ S	37°27′ E	1504	Optimal	58 P, 120 N	684	641	14.3, 24.1	14.7, 24.9	7.39	4.48
Kakamega	Kenya	0°16′ N	34°49′ E	1585	Optimal	37 P, 93 N	1449	1243	14.3, 26.8	14.4, 27.5	6.03	7.91
					Low N stress	85 P, 0 N					3.03	4.81
Namulonge	Uganda	0°32′ N	32°35′ E	1150	Optimal	27 P, 77 N	547	435	15.8, 27.6	16.6, 28.8	5.49	4.01
Bako	Ethiopia	9°12′ N	37°08′ E	1650	Optimal	45 P, 100 N	1151	875	13.7, 26.6	11.8, 27.3	6.30	8.34
					Low N stress	45 P, 0 N					3.20	2.28
Kiboko	Kenya	2°15′ S	37°75′ E	975	Managed drought stress	60 P, 87 N	71†	45†	16.0, 30.0	16.4, 30.1	0.42	1.34
Chiredzi	Zimbabwe	21°02′ S	31°58′ E	433	Managed drought stress	56 P, 120 N	0	0	12.4, 30.1	–	1.34	–

† Rainfall received in November just before harvest of trials.

### Drought and Low-Nitrogen Stress Management

Trials were planted at Kiboko in Kenya (2008 and 2009) and Chiredzi in Zimbabwe (2008) under managed drought stress during the rain-free period (June–October) at both locations. Irrigation water was applied using sprinklers at planting to establish a good plant stand and at regular intervals to avoid water stress during vegetative growth. Irrigation water in these trials was withdrawn 30 (V12 stage) and 45 d (V15 stage) after planting at Chiredzi and Kiboko, respectively. Total irrigation water applied from planting to the time of stopping water supply was ~260 mm at Kiboko and 220 mm at Chiredzi. This water withdrawal led to severe drought stress (mean yield = 15–20% of well-watered yield) in the trials, as the germplasm in this study was of the intermediate to late maturity category. Additional details on drought stress management are provided in Bänziger et al. (2000). The trials under managed drought stress received P at planting and N fertilizer as topdressing according to recommended rates for each location (Table 1). Four trials (two each at Kakamega in Kenya and Bako in Ethiopia) were planted under managed low N stress conditions. The fields used for low-N trials had previously been depleted of N by growing maize continuously without applying N fertilizer and removing crop biomass after each season for at least 4 yr, following the guidelines described by Bänziger et al. (1997, 2000). For trials planted under managed low N stress, P fertilizer was applied at planting, but no N fertilizer was applied as topdressing. At harvest, ears from plants at each end of a row in both drought-stressed and low-N-stressed trials were discarded because they experienced less competition and greater access to water available in the alleys between blocks in a trial.

### Data Collection

Data were recorded on agronomic traits and field ear weight on a plot basis in the trials. Data on the following traits were recorded: days to anthesis (AD, recorded as days from planting to when 50% of the plants started to shed pollen), ear height (EH, measured in centimeters as the distance from the base of the plant to the point of attachment of the top ear on a plant), plant height (PH, measured in centimeters as the distance from the base of the plant to the base of the first tassel branch), husk cover (HC, measured as percentage of plants with ears not completely covered by the husks), and number of ears per plant (EPP, determined by dividing the total number of ears per plot by the number of plants harvested per plot). All ears harvested from each two-row plot were weighed, and representative samples from shelled ears were taken to determine percentage moisture using a Dickey-John multigrain moisture tester (Dickey-John Corporation) at all locations. Grain yield expressed as megagrams per hectare was calculated from shelled grain weight (in drought, low-N, and random stress trials) or ear weight in optimal trials where a shelling percentage of 80% was assumed and GY was adjusted to 12.5% moisture content.

### Simple Sequence Repeat Genotyping

Leaf samples were collected from greenhouse-grown seedlings at the three- to four-leaf stage for each of the seven OPVs for DNA extraction, which was done by bulking an equal amount of leaf tissue from each entry. Each OPV was represented by two bulks of 15 plants each. Details on DNA quality analysis and concentration were the same as described by Semagn et al. (2014). A set of 47 SSR markers, also used by Semagn et al. (2014), was used for genotyping (Supplemental Table S1). Briefly, polymerase chain reaction (PCR) was performed in 96-well plates in a total reaction volume of 10 μL that consisted of 30 ng DNA, 1× magnesium-free PCR buffer, 2 mM MgCl_2_, 0.20 μM of a forward primer labeled with 6-FAM, PET, VIC, or NED fluorescent dyes, 0.20 μM of a reverse primer, 0.20 mM of each deoxynucleotide, and 0.25 U AmpliTaq Gold DNA polymerase. The PCR amplifications were performed for each primer pair separately using a Gene-Amp PCR System 9600 (PE-Applied Biosystems) (Semagn et al., 2014). After the PCR, ~3 μL of the PCR product from four randomly selected samples per marker was checked for proper amplification and product intensity by running the samples on a 2% agarose gel. Additional details about PCR conditions and allele calling can be found in Semagn et al. (2014).

### Statistical Analyses

The data were tested for homogeneity of variance using Levene’s test before conducting ANOVA. Analyses of variance were performed using PROC MIXED of SAS (SAS Institute, 2011) on data adjusted for maturity (used as a covariate). Entries were considered fixed effects, whereas locations were considered random effects. The following linear model was used for combined analysis for each environment:

$$Y_{ijrk}=\mu +\alpha_{i}+\beta_{j}+\rho_{r}\left(\beta_{j}\right)+\lambda_{k}[\rho_{r}\left(\beta_{j}\right)]+{\alpha \beta }_{ij}+\epsilon_{ijrk}$$

where *Y_ijrk_* is the mean of the *i*th genotype in the *r*th replicate within the *k*th sub-block of the *j*th environment, μ is the grand mean, α*_i_* is the effect of the *i*th genotype, β*j* is the effect of the *j*th environment, ρ*_r_* is the effect of the *r*th replicate, ρ*_r_*(β*_j_*) is the effect of the replicates within environments, λ*_k_* is the effect of the *k*th incomplete block, λ*_k_*[ρ*_r_*(β*_j_*)] is the effect of the incomplete blocks within replicates and environments, αβ*_ij_* is the effect of genotype × environment interaction, and ε*_ijrk_* is the residual error. In the across-environments ANOVA, genotype effects were tested for significance using the corresponding interaction with the environment as the error term, whereas the genotype × environment interaction was tested against the pooled error. Each location-year combination was considered an environment. All factors were considered random effects to estimate variance components. Broad-sense heritability (*H*^2^) for traits across environments was estimated using variance components, according to Hallauer et al. (2010), as

$$H^{2}=\frac{\sigma_{\text{G}}^{2}}{\sigma_{\text{G}}^{2}+\frac{\sigma_{\text{GL}}^{2}}{e}+\frac{\sigma_{\text{E}}^{2}}{er}}$$

where $\sigma_{\text{G}}^{2},\sigma_{\text{GL}}^{2},\sigma_{\text{E}}^{2}$ are the genotype, genotype × location, and residual variance components, respectively; *e* is the number of environments; and *r* is the number of replications.

### Diallel Analysis

The data, excluding that of the checks, were subjected to Analysis II of Gardner and Eberhart (1966) for a population diallel according to the linear model

$$Y_{{jj}^{\prime }}=\mu_{\text{v}}+0.5\left(v_{j}+v_{j^{\prime }}\right)+v\overset{¯}{h}+v\left(h_{j}+h_{j^{\prime }}\right)+{vs}_{{jj}^{\prime }}$$

where Y*_jj_*_′_ is the mean of a parent when *j* = *j*′ and of a cross when *j ≠ j*′; μ_v_ is the mean of all varieties; *v_j_* and *v_j_*′ are variety effects for varieties *j* and *j*′, respectively, when they are included in the analyses; *h̄* is the average heterosis contributed by a particular set of varieties; *h_j_* and *h_j_*′ is the variety heterosis for varieties *j* and *j*′, respectively; *s_jj_*_′_ is the specific heterosis that occurs when varieties *j* and *j*′ are mated; and *v* = 0 when *j* = *j*′ and *v* = 1 when *j ≠ j*′.

Analysis III of Gardner and Eberhart (1966) was used to obtain estimates of general combining ability (GCA) effects of the populations. The linear model used for Analysis III is

$$Y_{{jj}^{\prime }}=\mu_{\text{v}}+v_{j}+v\overset{¯}{h}+{vx}_{{jj}^{\prime }}\text{ and }x_{{jj}^{\prime }}=g_{j}+g_{j^{\prime }}+s_{{jj}^{\prime }}$$

where Y*_jj_*′ is the mean of a parent when *j* = *j*′ and of a cross when *j* ≠ *j*′; μv is the mean of all varieties; *v* is the variety effect; *h̄* is the average heterosis; *x_jj_*′ is the cross effect for the mating between varieties *j* and *j*′; *g_j_* and *g_j_*′ are GCA effects for varieties *j* and *j*′, respectively, and *s_jj_*′ is the specific combining ability effect. Analyses II and III of Gardner and Eberhart (1966) were performed using DIALLEL-SAS05 software (Zhang et al., 2005).

### Selection Index and Heterosis

A base index (Williams, 1962), modified to incorporate both an assigned relative trait economic weight and heritability (Smith et al., 1981), was constructed and used to identify population hybrids suitable for utilization across the agroecological conditions in ESA. We assigned arbitrary relative economic weights according to the importance of the trait from a breeding standpoint and desirability by farmers in the region. A trait that is ranked highly by both breeders and farmers was assigned higher weight than other traits. In this index, a higher weight was assigned to GY performance under optimal conditions (4), followed by performance under managed drought (2.5), low N (1.5), and random abiotic stress (0.7) conditions. Other traits were assigned economic weights according to their relative importance, as shown in the expression below. The least-squares means for the traits included in the index were standardized, with mean = 0 and SD = 1. The base index score (*I*) for each entry with assigned relative economic weight for each trait was calculated in Microsoft Excel as

I=[(4×GY-O×H)+(2.5×GY-D×H)+(1.5×GY-L×H)+(0.7×GY-R×H)+(1.5×EPP×H)+(−0.1×AD×H)+(−0.1×PH×H)+(−0.1×EH×H)+(−0.5×HC×H)]

where GY-O, GY-D, GY-L, and GY-R are GY under optimal conditions, managed drought, managed low-N stress, and random abiotic stress, respectively, and *H* is broad-sense heritability of the respective trait. Mid-parent (MPH) and high-parent (HPH) heterosis for GY were calculated using the adjusted means of the population hybrids and their parental populations.

Mid-parent heterosis was calculated as

$$\text{MPH}=\frac{\left(F_{1}-\text{MP}\right)}{\text{MP}}\times 100$$

where *F*_1_ is the hybrid mean performance, and MP = (*P*_1_ + *P*_2_)/2, where *P*_1_ and *P*_2_ represent mean performance of Parent 1 and Parent 2, respectively.

High-parent heterosis was calculated as

$$\text{HPH}=\frac{\left(F_{1}-\text{HP}\right)}{\text{HP}}\times 100$$

where HP is mean performance of the high parent.

### Clustering of Population Hybrids and Parents

Adjusted mean GY from the 21 environments (10 optimal, four each of managed low N and random abiotic stress, and three managed drought stress) was standardized to a mean of zero and variance of one and subjected to cluster analysis. Ward’s (1963) minimum variance clustering method was used to group the population hybrids with similar performance. The SAS command PROC CLUSTER (SAS Institute, 2011) was used for cluster analysis. The PROC TREE command of SAS was used to generate a dendrogram. We calculated correlations between mean GY and AD, PH, EH, EPP, and HC under each of the four management conditions for the population hybrids and their parents. We then used the correlation matrix for conducting principal component analysis (PCA) by invoking the PROC PRINCOMP command of SAS (SAS Institute, 2011). The principal component (PC) scores for the first two axes (PC1 and PC2) were plotted to visualize the potential separation of the 28 genotypes into groups.

### Diversity Analysis

Relative allele frequency, Shannon information index, and the expected and unbiased expected heterozygosity were calculated using GenAlEx version 6.5 (Peakall and Smouse, 2012). Polymorphic information content (PIC) was calculated using the PIC calculator (https://www.liverpool.ac.uk/~kempsj/pic.html). Principal coordinate analysis (PCoA) based on the Rogers and Tanimoto (1960) similarity matrix was conducted via GenStat version 18 (VSN International, 2015). Genetic distance between pairs of populations was calculated according to Edwards (1971) as

$${\text{GD}}_{\left(A,B\right)}=\sqrt{1-\frac{1}{v}\overset{v}{\underset{k=1}{\Sigma }}\overset{m\left(k\right)}{\underset{j=1}{\Sigma }}\sqrt{p_{Ajk}p_{Bjk}}}$$

where *v* is the number of loci, *m* is the number of alleles, and *p_Ajk_* and *p_Bjk_* are the sums of all specific allele frequencies at a single locus for populations *A* and *B*, respectively. The GD was computed and a dendrogram based on Edward’s GD was constructed via the “poppr” package (Kamvar et al., 2014) in R. Pearson correlation coefficients between GD and heterosis were calculated using the PROC CORR command in SAS.

## RESULTS

### Analysis of Variance and Genetic Effects

The combined ANOVA across 10 optimal environments showed significant (*P* < 0.001) environment and entry mean squares for GY (Table 2) and other traits (Supplemental Table S2). Partition of the variation among generation means revealed that both variety (*v_j_*) and heterosis (*h_jj_*′) effects were significant for GY and AD. Heterosis accounted for 67 and 29% of the entry sum of squares for GY and AD, respectively. Partition of heterosis into three components showed that only *h̄* was significant for GY and accounted for 86% of the entry sum of squares for heterosis. The entry × environment, *v_j_* × environment, and *h̄* × environment interactions were significant for GY.

**Table 2:** Mean squares from combined Gardner and Eberhart (1966) Analysis II of seven maize populations and their diallel crosses evaluated under four management options and across environments over 2 yr (2008 and 2009).

Source of variation	Optimal		Managed drought		Managed low N		Random abiotic stress		Across environments	
	df	Grain yield	df	Grain yield	df	Grain yield	df	Grain yield	df	Grain yield
Environments (E)	9	288.31***	2	18.17***	3	100.36***	3	91.32***	20	419.10***
Replications/E	18	6.39***	4	0.89*	8	5.16***	8	4.18***	38	5.01***
Entries	27	9.34***	27	0.48**	27	4.73***	27	1.12*	27	10.62***
Varieties (*v_j_*)	6	14.49***	6	1.39*	6	13.23**	6	2.61**	6	21.41***
Heterosis (*h_jj′_*)	21	8.33***	21	0.45**	21	2.30***	21	0.70	21	8.40***
Average heterosis ($\overset{¯}{h}$)	1	150.43***	1	1.54	1	34.71*	1	2.88**	1	147.13***
Variety heterosis (*h_j_*)	6	1.60	6	0.38*	6	0.83	6	0.62	6	2.33**
Specific heterosis (*s_jj′_*)	14	1.06	14	0.40*	14	0.61	14	0.58	14	1.09
E × Entries	243	1.24*	54	0.22	81	0.80	81	0.62	540	1.03***
E × *v_j_*	54	1.95***	12	0.45	18	1.48**	18	0.61	120	1.69***
E × *h_jj′_*	189	1.04	42	0.17	63	0.60	63	0.63	420	0.87
E × $\overset{¯}{h}$	9	4.61***	2	0.59	3	0.68	3	0.05	20	4.52***
E × *h_j_*	54	0.78	12	0.09	18	0.57	18	0.65	420	0.58
E × *s_jj′_*	126	0.89	28	0.17	42	0.61	42	0.66	280	0.73
Pooled error	486	0.98	101	0.26	216	0.70	216	0.75	1017	0.80

*,**,*** Significant at the 0.05, 0.01, and 0.001 probability levels, respectively.

Across managed drought stress conditions, significant differences among populations for GY were detected (Table 2). Both additive (*vj*) and nonadditive (*h_jj′_*) genetic effects were significant for GY. Heterosis effects explained 53, 68, and 88% of the entry sum of squares for GY, EPP, and AD, respectively, whereas specific heterosis accounted for 59% of the entry sum of squares for heterosis for GY under managed stress. Across low-N-stress conditions, significant variety and heterosis effects for GY and AD were detected. Average heterosis explained 72% of the entry sum of squares for heterosis for GY under low-N stress. Across random abiotic stress conditions, significant differences existed among entries for GY. The variety effect was significant for GY and explained 52% of the entry sum of squares under random abiotic stress. Average heterosis was the only significant component of heterosis.

The combined ANOVA across all environments showed highly significant (*P* < 0.001) differences among environments and entries for all traits (Table 2, Supplemental Table S2). Partition of variation among generation means showed that *v_j_* and *h_jj_*′ were significant for GY and AD. Heterosis accounted for 58% of the entry sum of squares for GY. Average heterosis accounted for 83 and 64% of the entry sum of squares for heterosis for GY and AD, respectively. The entry × environment, *v_j_* × environment, and *h̄* × environment interactions were significant for GY.

The genetic effects (variety, heterosis, and GCA) for traits varied among populations and environments (Table 3, Supplemental Table S3). Population ECAVL2 had the highest variety effect (*v_j_*) for GY under managed drought, under low N, and across environments (Table 3). Populations ECAVL16-STR and ECAVL18 had positive variety effect for GY under managed low N. Populations ECAVL2 and ECAVL18 had positive variety effects for GY under all conditions and across environments. The variety heterosis (*h_j_*) estimate for GY was consistently positive for population ECAVL17 across stress and nonstress conditions, as well as across environments. Average heterosis was significantly different from zero for GY under optimal and low-N conditions, which indicated that the mean of the population hybrids was higher than the mean of the parental populations. Population ECAVL2 had a significant positive GCA effect for GY under managed low-N conditions and across environments (Table 3). Population NIP25 had the smallest GCA effects for PH (Supplemental Table S3) and produced hybrids with shorter plants compared with hybrids between other populations (data not shown).

**Table 3:** Estimates of variety effects (*v_j_*), variety heterosis (*h_j_*), general combining ability (GCA) effects, and variety mean for grain yield under three management options and across environments over 2 yr (2008 and 2009).

Population	Optimal conditions				Managed drought				Managed low N				Across environments			
	*v_j_*	*h_j_*	GCA	Mean	*v_j_*	*h_j_*	GCA	Mean	*v_j_*	*h_j_*	GCA	Mean	*v_j_*	*h_j_*	GCA	Mean
	———————————————————————————————————————— Mg ha^–1^ ————————————————————————————————————————															
ECAVL1	−0.11	−0.05	−0.10	5.07	0.04	0.06	0.08	0.82	−0.05	−0.06	−0.04	2.98	−0.02	−0.03	−0.03	3.51
ECAVL2	0.51	0.02	0.28	5.77	0.65*	−0.17	0.16	1.61	0.96**	−0.02	0.46**	3.69	0.54	0.03	0.30*	4.09
ECAVL16	−0.66	−0.10	−0.43*	4.44	−0.09	−0.27	−0.31**	0.71	−1.15**	−0.14	−0.71***	1.71	−0.47	−0.19	−0.43**	2.93
ECAVL16-STR	0.40	−0.20	0.00	5.52	−0.18	−0.02	−0.11	0.81	0.33	−0.26	−0.09	3.24	0.15	−0.13	−0.05	3.70
ECAVL17	−0.46	0.32	0.09	4.74	−0.38	0.13	−0.06	0.47	−0.05	0.22	0.20	2.89	−0.35	0.20	0.03	3.21
ECAVL18	0.59	0.00	0.30	5.82	0.14	0.13	0.20	1.13	0.24	0.22	0.33	3.14	0.27	0.10	0.24	3.87
NIP25	−0.27	0.01	−0.13	4.85	−0.18	0.14	0.05	0.62	−0.37	0.04	−0.15	2.45	−0.13	0.02	−0.04	3.32
SE/LSD_0.05_^†^	0.38	0.25	0.17	0.56	0.25	0.17	0.11	0.51	0.36	0.24	0.16	0.72	0.30	0.20	0.13	0.35
Average heterosis		1.01***				0.21				0.74***				0.69***		

*,**,*** Significant at *P* < 0.05, *P* < 0.01, and *P* < 0.001, respectively.

† SE of variety, heterosis and GCA effects, LSD for mean grain yield.

### Performance, Heterosis, and Selection

The highest yielding population under optimal conditions was ECAVL18, whereas that under managed stress conditions and across environments was ECAVL2 (Table 3). Grain yield ranged from 5.5 to 6.8 Mg ha^–1^ under optimal conditions for the population hybrids (Table 4). Populations ECAVL2, ECAVL18, and ECAVL16-STR were parents of most of the top-yielding hybrids under optimal conditions. Population ECAVL16-STR produced high-yielding hybrids when crossed with populations ECAVL2 and ECAVL18, and these hybrids performed better than similar hybrids with population ECAVL16 as the second parent in most cases. Under managed low N, GY ranged from 2.8 to 4.4 Mg ha^–1^, whereas under managed drought stress, GY ranged from 0.7 to 1.7 Mg ha^–1^. The population hybrids, on average, yielded 43 and 82% less under low N and managed drought, respectively, compared with optimal conditions. Across environments, GY was highest (4.8 Mg ha^–1^) for population hybrid ECAVL2 × ECAVL18.

**Table 4:** Mean grain yield and mid- (MPH) and high-parent heterosis (HPH) for grain yield of 21 population hybrids evaluated under optimal, managed drought stress, managed low N, and across environments for 2 yr (2008 and 2009).

Entry	Pedigree	Optimal conditions			Managed low N			Managed drought				Across environments				Base Index	
		Grain yield	MPH	HPH	Grain yield	MPH	HPH	Grain yield	MPH	HPH	Specific heterosis	Grain yield	MPH	HPH	Specific heterosis	Value	Rank
		Mg ha^–1^	—— % ——		Mg ha^–1^	—— % ——		Mg ha^–1^	—— % ——			Mg ha^–1^	—— % ——				
1	ECAVL1 × ECAVL2	6.3	16	9	3.9	17	7	1.3	11	−16	−0.04	4.4	16	8	−0.03	5.1	4
2	ECAVL1 × ECAVL16	5.7	21	13	3.2	25	3	0.8	1	−6	−0.18	3.9	21	11	0.04	−8.5	20
3	ECAVL1 × ECAVL16-STR	6.0	13	9	3.5	10	7	1.3	62	61	0.19	4.2	16	13	−0.05	−2.1	14
4	ECAVL1 × ECAVL17	5.9	20	16	3.5	16	15	1.3	101	58	0.18	4.1	21	15	−0.19	−2.7	15
5	ECAVL1 × ECAVL18	6.4	18	11	3.7	18	15	1.3	37	18	−0.07	4.4	20	15	0.07	2.1	9
6	ECAVL1 × NIP25	6.2	24	22	3.4	19	11	1.0	42	25	−0.09	4.2	22	19	0.17	−0.5	11
7	ECAVL2 × ECAVL16	6.1	20	6	3.5	23	−4	1.0	−17	−40	−0.05	4.3	21	4	0.08	−1.5	13
8	ECAVL2 × ECAVL16-STR	6.7	20	17	3.8	11	6	1.2	0	−25	0.01	4.6	19	14	0.04	7.0	3
9	ECAVL2 ´ ECAVL17	6.6	25	14	4.0	20	9	1.2	16	−25	−0.05	4.6	27	13	0.05	4.6	5
10	ECAVL2 × ECAVL18	6.8	18	17	4.4	29	21	1.6	17	0	0.08	4.8	22	18	0.02	14.0	1
11	ECAVL2 × NIP25	6.1	14	5	3.5	12	−3	1.2	9	−25	0.05	4.3	15	4	−0.16	−0.6	12
12	ECAVL16 × ECAVL16-STR	5.5	11	1	3.1	18	−4	1.0	32	24	0.11	3.7	13	1	−0.08	−8.4	19
13	ECAVL16 × ECAVL17	6.1	32	28	3.0	21	1	1.0	66	39	0.19	4.0	31	25	0.10	−3.9	16
14	ECAVL16 × ECAVL18	6.1	18	4	3.1	18	−4	0.8	−14	−30	−0.33	4.0	16	2	−0.12	−6.6	17
15	ECAVL16 × NIP25	5.6	20	15	2.8	19	6	1.1	65	55	0.26	3.7	19	12	−0.03	−9.5	21
16	ECAVL16-STR × ECAVL17	6.4	25	16	3.9	25	20	1.1	66	32	0.11	4.4	27	19	0.13	3.3	8
17	ECAVL16-STR × ECAVL18	6.8	19	16	3.6	12	10	1.2	19	2	−0.01	4.5	20	17	0.13	3.7	7
18	ECAVL16-STR × NIP25	6.0	16	9	3.2	8	−2	0.7	−4	−15	−0.42	4.0	15	9	−0.17	−6.6	18
19	ECAVL17 × ECAVL18	6.4	22	11	3.9	26	22	1.2	45	3	−0.15	4.4	24	13	−0.19	3.9	6
20	ECAVL17 × NIP25	6.4	33	32	3.6	28	21	0.9	71	51	−0.28	4.4	33	31	0.10	−0.1	10
21	ECAVL18 × NIP25	6.2	16	6	4.0	36	24	1.7	97	53	0.48^*^	4.4	23	14	0.09	7.3	2
WH403 (check hybrid)		7.8			3.0			1.5				5.4					
Mean		5.8			3.4			1.2				4.1					
LSD_0.05_		0.78			0.83			0.44			0.22†	0.46			0.26†		
Heritability		0.86			0.77			0.68				0.92					

* Significant at the 0.05 probability level.

† Standard error.

Both MPH and HPH varied under the different conditions. Mid-parent heterosis for GY ranged from 11 to 33% under optimal conditions, 8 to 36% under low N, and −17 to 101% under managed drought stress. Mid-parent heterosis was highest for population hybrids ECAVL18 × NIP25 and ECAVL1 × ECAVL17 under low N and managed drought stress, respectively. Average MPH for GY was highest under managed stress compared with low-N and optimal conditions. Of the 21 crosses, 17 showed positive MPH under managed drought-stress conditions. High-parent heterosis under optimal conditions ranged from 1 to 32% and was highest for the ECAVL17 × NIP25 cross. Under low N and managed drought stress, HPH was highest for crosses ECAVL18 × NIP25 (24%) and ECAVL1 × ECAVL16-STR (61%), respectively. Average HPH for GY was similar across all conditions and environments. Four crosses exhibited negative HPH under both low N and drought stress, whereas three crosses showed negative HPH under managed drought stress. Five crosses (ECAVL1 × ECAVL16-STR, ECAVL1 × ECAVL17, ECAVL16 × NIP25, ECAVL17 × NIP25, and ECAVL18 × NIP25) with high MPH (>50%) also had high HPH under managed drought stress conditions. Under optimal conditions, AD ranged from 76 to 79 d for the populations and 76 to 78 d for the population hybrids (Supplemental Tables S3 and S4). The genotypes took longer to reach anthesis and were shorter under stress environments than under optimal conditions (Supplemental Table S4). The EPP was lowest under managed drought-stress conditions.

**Fig. 1:**
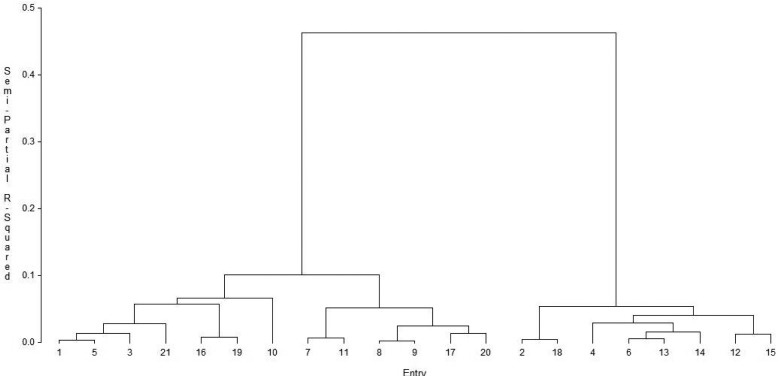
Dendrogram of 21 population hybrids based on grain yield across 21 environments (optimal, managed drought, low N, and random abiotic stress) using Ward’s minimum variance method. Entry pedigrees are provided in Table 4.

**Fig. 2:**
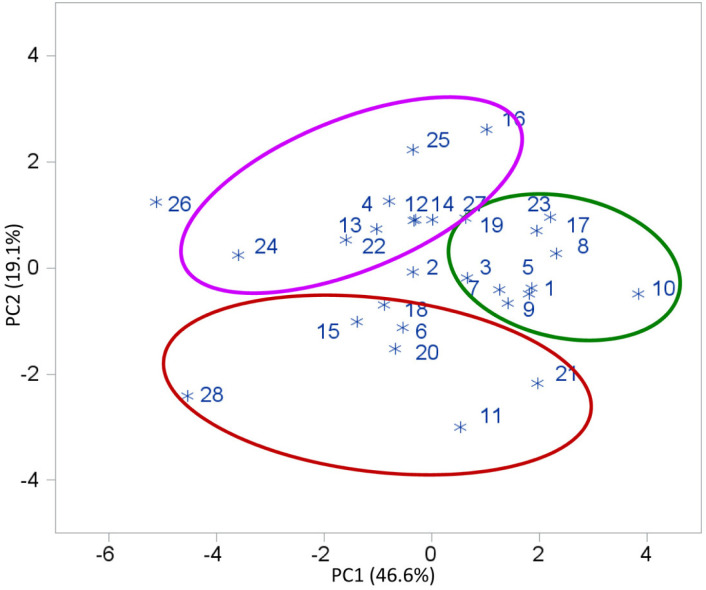
Principal component analysis of grain yield and five agronomic traits of 21 population hybrids and their parents under four management regimes (optimal, managed drought, low N, and random abiotic stress) across 21 environments in 2008 and 2009. Pedigrees of the population hybrids (Entries 1–21) are given in Table 4. Parental populations: 22 = ECAVL1, 23 = ECAVL2, 24 = ECAVL16, 25 = ECAVL16-STR, 26 = ECAVL17, 27 = ECAVL18, and 28 = NIP25. PC1 and PC2 refer to Principal Components 1 and 2 , respectively.

A selection index was used to identify population hybrids that combined good performance relative to GY across a range of conditions with desirable agronomic traits. Results showed that population hybrid ECAVL2 × ECAVL18 had the largest index value (14.0), followed by ECAVL18 × NIP25 (7.3) (Table 4). Five population hybrids (Entries 1 [P1 × P2], 8 [P2 × P4], 9 [P2 × P5], 10 [P2 × P6], and 17 [P4 × P6]) had positive index values for GY under all conditions (data not shown). Cluster analysis of the 21 population hybrids based on GY performance across 21 environments revealed two major clusters (Fig. 1). Cluster I consisted of 13 population hybrids that were divided into two subgroups. The population hybrids in Subgroups I and II were predominantly the top-yielding hybrids, with the majority of them having positive index values, except four entries (3, 7, 11, and 20). Subgroup I consisted of hybrids with higher GY under both managed stress conditions than those in Subgroup II. Cluster II consisted of eight population hybrids with lower yield, on average, than those in Cluster I, and all had negative index values. Principal component analysis revealed that the first two PCs accounted for 65.7% of the total variation (Table 5). The first PC was strongly associated with GY (under optimal, managed drought, and low-N conditions), PH, EH, and EPP. The second PC had higher loadings on AD and HC. A plot of the two PCs showed separation of the genotypes into three potential groups (Fig. 2). One group comprised Population NIP25 (Entry 28) and hybrids in which it was one of the parents. A second group comprised Population ECAVL2 (Entry 23) and hybrids in which this population was a parent along with other hybrids. A third group had the rest of the populations and hybrids.

**Table 5:** Eigenvectors of the first two principal component axes (PC1 and PC2) based on a correlation matrix of grain yield and other agronomic traits across 21 environments (2008–2009).

Traits	PC1	PC2
Grain yield under random abiotic stress (Mg ha^–1^)	0.243	−0.475
Grain yield under optimal conditions	0.424	−0.049
Grain yield under managed drought stress	0.401	−0.142
Grain yield under managed low N	0.415	−0.018
Days to anthesis	0.148	0.408
Plant height (cm)	0.380	0.328
Ear height (cm)	0.331	0.257
Ears per plant (no.)	0.390	−0.144
Husk cover (%)	−0.021	0.625
Proportion of variance explained (%)	46.6	19.1

### Genetic Diversity and Relationship with Heterosis

**Fig. 3:**
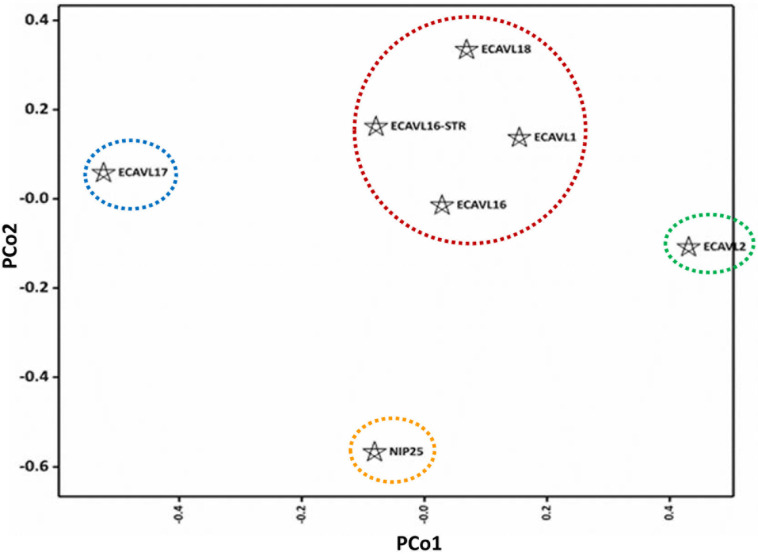
Principal coordinate analysis of seven parental populations using 47 simple sequence repeat markers. The first (PCo1) and second (PCo2) principal coordinates accounted for 20.1 and 19.8% of the variation, respectively.

**Fig. 4:**
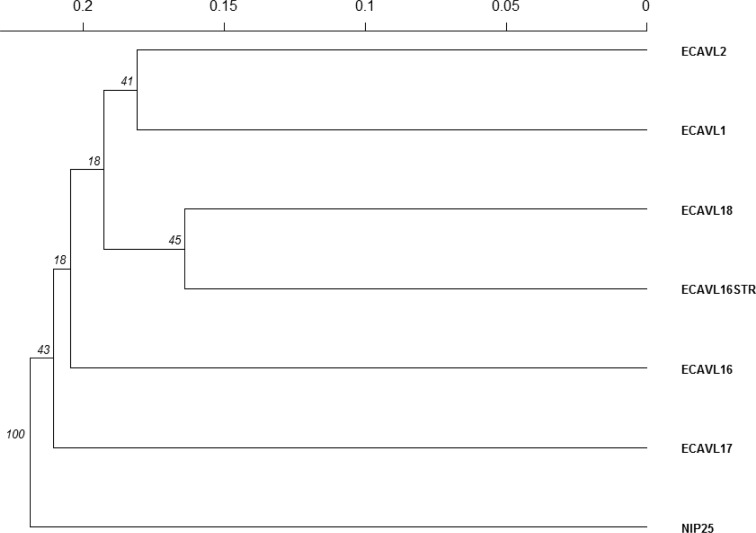
Dendrogram of seven parental populations based on Edwards (1971) genetic distance calculated from 47 simple sequence repeat markers. Numbers near the joints are bootstrap values for the dendogram clade.

The genetic diversity of parental populations was examined using 47 SSR markers, with the number of alleles per SSR varying from one to eight (Supplemental Table S1). The mean expected heterozygosity was 0.36, with a range of 0.14 to 0.50 (Table 6). Estimates of unbiased expected heterozygosity had minimal deviation from expected heterozygosity. Heterozygosity represents gene diversity; hence, some loci had a substantial degree of similarity, whereas others had wider diversity for SSR markers used in this study. Shannon’s allele information index ranged from 0.26 to 0.69, which agreed with expected heterozygosity. Mean PIC was 0.28. The PCoA results showed that the first (PCo1) and second (PCo2) coordinates explained 20.1 and 19.8% of the variation, respectively (Fig. 3). According to the similarity matrix, four populations (ECAVL1, ECAVL16, ECAVL16-STR, and ECAVL18) could be considered one cluster, although they were not very close, whereas NIP25 was the most distantly related. Populations ECAVL17 and ECAVL2 were nearly equidistant from the main cluster but further apart from each other. The GD estimates among pairs of populations ranged from 0.328 to 0.477 and averaged 0.404 (Table 7). The smallest GD was between populations ECAVL16-STR and ECAVL18, whereas the largest GD was between populations ECAVL2 and ECAVL17. Cluster analysis based on the Rogers and Tanimoto (1960) similarity matrix was consistent with PCoA results, where populations ECAVL17 and NIP25 were differentiated from other populations (Fig. 4). In the dendrogram, populations ECAVL2 and ECAVL1 were grouped together, although the two were not very close according to PCoA clustering. In the present study, most of the highest yielding hybrids involved parents from different clusters. For example, under optimal conditions, seven out of the top nine hybrids (yield range = 6.3–6.8 Mg ha^–1^) were crosses between parents from different clusters (Table 4, Fig. 3). Similarly, under managed drought stress, 8 out of the 11 top yielding hybrids (GY = 1.2–1.7 Mg ha^–1^) had parents from different clusters. A similar trend was evident under low N. The grouping of parental populations based on SSR markers and GY revealed similar patterns (Fig. 2 and 3). The correlation between GD and heterosis was low and ranged from 0.14 to 0.40 under optimal and stress conditions (Supplemental Table S5).

**Table 6:** Estimates of diversity parameters and polymorphic information content calculated from 47 simple sequence repeat markers used to genotype seven populations.

Estimates	Avg.	Min.	Max.
Shannon’s information index	0.53	0.26	0.69
Expected heterozygosity	0.36	0.14	0.50
Unbiased expected heterozygosity	0.38	0.15	0.54
Polymorphic information content	0.28	0.13	0.37

**Table 7:** Genetic distance estimates between seven populations calculated according to Edwards (1971).

Population	ECAVL1	ECAVL2	ECAVL16	ECAVL16STR	ECAVL17	ECAVL18
ECAVL2	0.352					
ECAVL16	0.453	0.406				
ECAVL16STR	0.367	0.375	0.398			
ECAVL17	0.398	0.477	0.406	0.359		
ECAVL18	0.367	0.422	0.406	0.328	0.438	
NIP25	0.414	0.422	0.406	0.406	0.438	0.438

## DISCUSSION

Improved populations are developed to increase the frequency of favorable alleles for improved GY performance and other specific attributes and are important components in the development of population hybrids that can be used in some communities where seed of three-way, double-cross, or single-cross hybrids is not readily available. This study was performed across a range of conditions and stresses commonly encountered by a large proportion of smallholder farmers in SSA. The significant population × environment interaction for GY and other traits observed in this study was because of the diverse germplasm and set of locations in four countries and management conditions used in this study. Variety effects for GY explained most of the variability among populations and their hybrids under low N and random abiotic stresses in this study, which suggested an important contribution of additive effects in the inheritance of GY under these stress conditions. This result is similar to findings in population diallel studies (Miranda Filho and Vencovsky, 1984; Pérez-Velásquez et al., 1995; Doerksen et al., 2003) conducted under optimal conditions but in contrast with findings in which nonadditive gene action was more important than additive gene action in the inheritance of GY under low N (Betrán et al., 2003a; Worku et al., 2008; Makumbi et al., 2011). These differences might be attributable to variation in the severity of low-N stress in the fields used in various studies, as different genetic mechanisms could be operating under different levels of N stress (Eisen and Saxton, 1983; Bänziger et al., 1997).

Heterosis effects for GY under optimal conditions, drought stress, and across environments in this study accounted for the majority of variation among generation means, which suggested that dominance effects played a major role in the inheritance of GY. These results corroborate other reports in population diallels (Mickelson et al., 2001; Doerksen et al., 2003; Soengas et al., 2003; Ron Parra et al., 2010) but are contrary to findings by Miranda Filho and Vencovsky (1984) and Crossa et al. (1990). Although this study and most other studies cited here used original populations, Doerksen et al. (2003) used advanced cycles of populations that had undergone recurrent selection. Reciprocal recurrent selection (RRS) improves performance and expression of heterosis (Hallauer, 1985; Hallauer et al., 2010). The differences between these results and those from other studies suggested that gene action controlling inheritance of GY and other agronomic traits in maize varied with germplasm and management conditions used. The presence of heterosis in all crosses and superiority of the crosses over the mid-parent values was apparent in this study, as indicated by the significant average heterosis for GY under optimal, low-N, and random abiotic stress conditions. The lack of significant specific heterosis for GY under low-N conditions suggested that the populations contributed similarly to crosses. This was expected, because additive genetic effects were of greater importance than nonadditive genetic effects in the inheritance of GY under low N in this study.

The populations with the highest favorable GCA effects for GY (ECAVL2 and ECAVL18) under most conditions in this study were parents to 73% of the top-yielding hybrids under optimal conditions, and 82% of the top-yielding hybrids under both low-N and managed drought conditions. This suggested that these two populations were good sources of alleles for GY and could be used to produce high-yielding population hybrids in combination with populations from other programs and for extraction of inbred lines. The broad genetic base of population ECAVL2 (78 component inbred lines) makes it a good candidate for extraction of inbred lines. Population ECAVL2 was developed using lines extracted from populations that had undergone improvement for various traits through several cycles of recurrent selection at CIMMYT (CIMMYT, 1998), and this probably contributed to its good per se performance and in hybrid combinations under both stress and nonstress conditions. Two populations (ECAVL2 and ECAVL18) that contained germplasm from Tuxpeño Sequía, a population improved for drought tolerance (Edmeades et al., 1999), were parents of hybrids with good performance under managed drought-stress conditions. The correlation between GY performance under managed low N and drought stress was strong (*r* = 0.703, *P* < 0.001), suggesting the presence of hybrids with good performance under both stress conditions. The implication is that breeders could use managed drought as an indirect selection environment for hybrids for low-N conditions. Since the establishment of low N screening sites poses challenges, the use of managed drought screening as an indirect selection environment becomes important. Bänziger et al. (1999) concluded that selection for drought tolerance may lead to morphological and physiological changes that are beneficial to maize when planted under N-stress conditions.

The results showed that the best populations to use in a RRS program to improve GY would be ECAVL2 and ECALV18 because of the favorable variety effects, positive GCA effects, and good per se performance under most of the conditions used in this study. If the objective of a maize breeding program was developing early-maturing material, the best two populations for RRS would be ECAVL16 and NIP25. A breeder selecting parents for recurrent selection for GY might consider populations ECAVL17 and ECAVL16, which had favorable variety effects for other traits under some conditions, in addition to populations ECAVL2 and ECALV18 that we have suggested for inclusion in a RRS program. To improve the combining ability of these populations in a recurrent selection scheme, the use of inbred line testers is recommended. With the reduced cost of genotyping, a marker-assisted recurrent selection scheme could be used to improve these adapted selected populations. In this case, inbred lines would be extracted from the populations and selected for important adaptive traits in the region (MSV, GLS, NCLB, and ear rots) using molecular markers. Early or advanced generations of the selected lines would then be recombined to form advanced cycles of these populations. Because of their adaptation to ESA, four populations (ECAVL2, ECALV17, ECAVL18, and ECALV16) would also be good candidates to be improved for *Striga hermonthica* (Delile) Benth. resistance using donor germplasm from IITA. *Striga* is a major biotic constraint to maize production in ESA, but resistant or tolerant germplasm adapted to the region is not yet widely available. Improvement of populations ECALV17 and ECAVL18 for resistance to *Striga* would be a good breeding objective, as these two populations, in combination with population ECALV16-STR, produced hybrids that had good performance under various conditions.

The average MPH recorded for GY under managed drought stress (34%) was comparable with that reported by Welcker et al. (2005) for maize populations under acid soils. Mid-parent heterosis was highest under stressed environments vs. optimal conditions, and these results are comparable with findings in other studies that used inbred lines (Betrán et al., 2003b; Makumbi et al., 2011). The MPH for GY was positive under optimal and low-N conditions, which suggested dominance or partial dominance of favorable alleles for GY under these conditions. Two populations, ECAVL17 and NIP25, contributed to high MPH in most of their crosses with other populations under optimal and stress conditions, possibly because of their lower per se yield, but it might also reflect the higher average diversity of these two populations than that of the other populations in this study. Population NIP25 was distantly related to the other six populations, and this could be explained by its origin from a single source population (Population 25) that contributed a few lines in the development of ECAVL2, but not any other population. However, Moll et al. (1965) indicated that crosses between extremely divergent populations might have limited heterosis. In this study, we recorded a 43% yield reduction under low N, which was lower than the 64% reported by Worku et al. (2008) and the 54% reported by Bänziger et al. (1997). The differences between the results in this study and other studies were probably attributable to different germplasm used and different stress levels imposed.

The best population hybrids were crosses between broad-based populations and synthetics. Some of the high-yielding population hybrids, such as ECAVL2 × ECAVL18 and ECAVL2 × ECAVL17, which also showed good heterosis, can be used to create new OPVs by advancing them to F_2_ through sib mating. The superior performance of Population ECAVL2 per se and in hybrid combinations makes it a good choice for further improvement. We have extracted inbred lines with good GY potential and disease resistance from segregating populations derived from crosses ECAVL2 × ECAVL17, ECAVL2 × ECAVL18, ECAVL2 × NIP25, and ECAVL1 × ECAVL2, which have been used to develop new stress-tolerant hybrids (CIMMYT, unpublished data, 2015). This suggested that many good yield–allele combinations were accumulated in the broad-based population ECAVL2 during its development. Extraction of inbred lines from population hybrids may offer an alternative way for small maize breeding programs in some countries to develop inbred lines, as opposed to using elite × elite inbred line F_2_s for inbred line development. With extraction of inbred lines from population hybrids formed using improved populations, there is a possibility that inbreeding depression, which hampers inbred line development from local OPVs, will be minimized. Inbred lines extracted from populations such as ECAVL2 that produced high-yielding population hybrids could be used for the prediction of potential superior F1 hybrids (Toledo and Miranda Filho, 1985). In addition, a population such as ECAVL2 could be used to transfer favorable alleles to improve other populations (Dudley, 1988). Given the good genetic effects of parents and performance of various hybrid combinations, ECAVL2 and ECAVL18 were the best female parents for use in nonconventional population hybrids among these populations. From a practical breeding standpoint, the results of heterosis recorded among the populations in this study indicate the potential of hybrid development to exploit heterosis. From two high-yielding but genetically distant populations, (e.g., ECAVL2 and ECAVL18), one could expect to extract inbred lines that contribute useful but different alleles for GY to produce superior hybrids suitable for different conditions when crossed. Furthermore, inbred lines extracted from such populations could be used to develop new narrow-based heterotic synthetics, which could be used as reservoirs of unique allelic combinations or as testers.

The results of PCA that showed lower loading for GY under random abiotic stress vs. other conditions justified our decision to assign a lower relative economic weight to GY under random abiotic stress. Earlier studies have indicated that selection is most efficient under managed stress environments, as opposed to random abiotic stress environments (Byrne et al., 1995; Weber et al., 2012). A selection index that incorporated heritability and assigned relative economic weights proposed by Smith et al. (1981) was useful in identifying top-yielding population hybrids that also combined good plant type and key agronomic traits. Indeed, some of the population hybrids with good index values have been released and are currently commercially grown in eastern Africa. For example, the population hybrid ECAVL2 × ECAVL18 with the highest index value was released in Uganda as ‘UH5053’ (MAAIF, 2016), and in Tanzania as ‘NATAH104’ (MAFSC, 2016) in 2012 and 2013, respectively. Additionally, two other population hybrids with positive index values (ECAVL1 × ECAVL18 and ECAVL2 × ECAVL17) were released in Uganda as ‘UH5051’ and ‘UH5052’, respectively (MAAIF, 2016). This shows that it is possible to develop and identify high-yielding population hybrids using improved populations. Such population hybrids are suitable for use as low-cost hybrids for a range of growing conditions and stresses in marginal areas of ESA with large proportions of smallholder maize farmers, who might not readily have access to hybrid seed. As noted by Pixley (2006), improved maize varieties that suffer less inbreeding depression on recycling are suitable for marginal environments where the market does not attract investment. Several small- and medium-sized seed companies have taken advantage of the improved population hybrids suitable for this region to provide improved seed to farmers. The rate of penetration of hybrid seed is still low in some areas, and population hybrids provide a better opportunity for small startup seed producers to bulk up adequate amounts of seed more quickly (fewer seasons compared with three-way hybrid seed production and lower technical demands), and their parental populations are easier to maintain. Such initiatives will be important in attaining increased maize productivity and production in the region. In the present study, population NIP25 had favorable GCA effects for PH, which suggested that it could be used as a source of alleles to reduce PH in midaltitude germplasm. This population is composed of inbred lines extracted from Population 25, which was earlier reported to produce short plants in hybrid combinations (Vasal et al., 1992).

Genetic diversity is essential for crop improvement, and the use of molecular markers to infer genetic diversity in maize is well documented (Xia et al., 2005; Wen et al., 2012; Semagn et al., 2014). We observed substantial diversity among parental populations, with a maximum unbiased expected heterozygosity of 0.54. This was supported by results from the Shannon information index, which has broad-spectrum applications and provides an estimate of genetic diversity in the context of population differentiation (Sherwin et al., 2006). The average PIC value recorded in this study was lower than those reported in several studies using tropical maize germplasm (Betrán et al., 2003b; Reif et al., 2003b; Xia et al., 2005). The lower average PIC value in this study could be attributed to the low average number of alleles per marker (2.3). The mean GD in this study (0.404) was higher than those reported by Semagn et al. (2014) for 218 OPVs from ESA (0.227) and by Reif et al. (2003b) for four tropical populations (0.241), but lower than that reported by Xia et al. (2005). The relatively large GD observed in this study could be attributed to the diverse background of the component lines used to develop the OPVs. For example, the two populations with the largest GD between them (ECAVL2 and ECAVL17) were of diverse backgrounds; ECAVL2 was composed of 78 lines, whereas ECAVL17 was composed of only eight lines. The presence of large GD among some of the populations suggested that they could be useful for further delineation of heterotic groups in tropical midaltitude maize germplasm. Heterotic groups are essential for hybrid and synthetic population development. For example, population ECAVL17 (Heterotic Group A), which was distantly related to most of the other populations, and population ECAVL18 (Heterotic Group B) could be used as testers for classification of populations and/or inbred lines into Heterotic Groups A and B, respectively. Proper classification of inbred lines and populations into well-defined heterotic groups ensures that hybrids developed using these lines or populations would maximize heterosis. The correlation between GD and heterosis was positive but low under both stress and optimal environments. This result is similar to findings in other studies that investigated genetic diversity and heterosis in maize (Betrán et al., 2003b; Dhliwayo et al., 2009; Makumbi et al., 2011) but contrary to findings by Reif et al. (2003a), who reported higher correlation (*r* = 0.63) between GD and heterosis in some tropical populations. Theoretical considerations have shown that a low correlation between GD and heterosis can be attributed to poor association between heterozygosity estimated from marker data and heterozygosity at quantitative trait loci (QTLs) affecting GY, and a poor association between heterozygosity and QTLs in the crosses studied (Charcosset et al., 1991).

There was good agreement between grouping of the populations based on phenotypic (GY and six other agronomic traits) and genotypic data. Populations ECAVL1, ECAVL16, and ECAVL16-STR were grouped in the same cluster in both SSR-based and phenotypic-based clustering, which confirmed similarity in the genetic constitution of these populations. These three populations had common ancestry (i.e., Population 43), whereas populations ECAVL16 and ECAVL16-STR had common parents from La Posta Sequía C3 and La Posta Sequía C7. In addition, both SSR-based and phenotypic clustering showed populations ECAVL2, ECAVL17, and NIP25 to be in different clusters and separate from the other clusters. This result suggested significant differentiation or allelic variation of these three populations. These populations could thus be good sources of allele combinations for many breeding programs interested in widening the genetic base of their germplasm. Indeed, crosses of populations ECAVL17 and NIP25 with other populations showed higher MPH in the majority of their crosses compared with other hybrid combinations. This result is important for breeding programs interested in developing hybrids using these populations or inbred lines extracted from these populations. There was a difference between PCoA clustering and grouping based on GD for populations ECAVL2 and ECAVL1. These two populations, with a broad genetic base, had some common ancestry, and this was reflected in the relatively small GD between the two that explained the close relatedness reflected in the dendrogram. Lack of agreement between two classification methods among OPVs has been reported in another study (Semagn et al., 2014). The information from SSR-based diversity and phenotypic analyses could be useful in defining breeding strategies and for maintenance of heterotic patterns among the populations used in this study.

## CONCLUSIONS

This study revealed that heterosis contributed greatly to variation among generation means for GY across managed drought stress, which indicated the important role of dominance effects. Populations with good genetic effects and performance were identified, and these could be used in a RRS program for further improvement of a number of important traits. There was agreement between GY-based and SSR-based clustering that showed three populations separated from the other populations, which suggested significant differentiation or allelic diversity of these three populations. There was relatively large GD among the populations that could be attributed to the diverse nature of the populations, and some of the populations in this study have the potential for use in heterotic group classification of ESA-adapted tropical maize OPVs. Several population hybrids between genetically distant populations that exhibited good performance across a range of conditions were identified and are marketed commercially as low-cost hybrids in the region.

## Conflict of Interest

The authors declare that there is no conflict of interest.

## Supplemental Material Available

Supplemental material for this article is available online.

## Abbreviations:

AD,: days to anthesis;
EH,: ear height;
EPP,: ears per plant;
ESA,: eastern and southern Africa;
GCA,: general combining ability;
GD,: genetic distance;
GLS,: gray leaf spot;
GY,: grain yield;
*h̄*,: average heterosis;
HC,: husk cover;
*h_ij_*_′_,: heterosis effects;
HPH,: high-parent heterosis;
KALRO,: Kenya Agricultural and Livestock Research Organization;
MPH,: mid-parent heterosis;
MSV,: *Maize streak virus*;
NCLB,: northern corn leaf blight;
OPV,: open-pollinated variety;
PC,: principal component;
PCA,: principal component analysis;
PCoA,: principal coordinate analysis;
PCR,: polymerase chain reaction;
PH,: plant height;
PIC,: polymorphic information content;
QTL,: quantitative trait locus;
RRS,: reciprocal recurrent selection;
SSA,: sub-Saharan Africa;
SSR,: simple sequence repeat;
*v_j_*,: variety effects.
