# Supplementary material for: Genetic Analysis of Tropical Midaltitude- Adapted Maize Populations under Stress and Nonstress Conditions
Source: Crop Sci. 2018 Jun 7;58:1492–507. doi: 10.2135/cropsci2017.09.0531 (PMC7680935; doi:10.2135/cropsci2017.09.0531)
Supplement: Supplementary file 1 [file CROPSCI-58-04-1492-s001.pdf]

Supplemental Table S1. List of 47 simple sequence repeat markers used to genotype the seven populations.

Marker #	Marker Name[†]	Bin	No. of polymorphic alleles	Marker #	Marker Name	Bin	No. of polymorphic alleles
1	phi109275	1.00	3	25	umc1332	5.04	1
2	phi056	1.01	3	26	phi085	5.07	3
3	umc1917	1.04	2	27	phi075	6.00	4
4	phi308707	1.10	3	28	umc1143	6.00	3
5	phi064	1.11	8	29	phi031	6.04	5
6	phi227562	1.12	4	30	phi123	6.07	1
7	phi96100	2.00	5	31	umc1545	7.00	1
8	nc133	2.05	2	32	phi112	7.01	1
9	phi090	2.08	1	33	phi114	7.02	3
10	phi127	2.08	1	34	phi051	7.05	2
11	phi104127	3.01	2	35	umc1304	8.02	1
12	phi453121	3.01	5	36	phi014	8.04	1
13	phi374118	3.02	5	37	umc1161	8.06	1
14	phi029	3.04	2	38	phi015	8.09	2
15	phi102228	3.04	2	39	phi108411	9.06	1
16	phi053	3.05	2	40	phi041	10.00	3
17	umc1136	3.10	6	41	phi063	10.02	3
18	phi072	4.01	3	42	phi059	10.02	2
19	phi079	4.05	4	43	umc1367	10.03	2
20	phi093	4.08	1	44	phi062	10.04	1
21	phi076	4.11	2	45	phi084	10.04	2
22	phi109188	5.00	2	46	umc1061	10.06	4
23	umc1447	5.03	3	47	umc1196	10.07	3
24	phi331888	5.04	4				

[†]Repeat motif, and annealing temperatures of the simple sequence repeat markers were provided in Semagn et al. (2014).

Supplementary Table 2. Mean squares from combined Gardner and Eberhart (1966) Analysis II of seven maize populations and their diallel crosses evaluated under three management conditions and across environments over two years (2008 and 2009).

	Optimal conditions				Managed drought			Managed low N				Across environments			
Source of variation	df[†]	EPP[‡]	AD[‡]	PH[‡]	df	AD	EPP	df	EPP	PH	AD	df	AD	PH	EPP
Environments (E)	9	1.95***	3663.05***	146495.12***	2	30098.55***	1.34***	3	0.45***	106070.04***	2606.29***	20	7564.49***	121928.12***	5.17***
Replications/E	18	0.07***	11.56***	1104.37***	4	1.86	0.05**	8	0.06	748.07**	9.59	38	10.26***	902.00***	0.07***
Entries	27	0.07***	24.94***	801.97**	27	7.00	0.01	27	0.03	821.90***	13.35***	27	37.71***	1166.75***	0.07***
Varieties (v_j)	6	0.26***	81.90***	1374.40*	6	5.12	0.02	6	0.04	2611.66***	37.03***	6	128.65***	3217.27***	0.26***
Heterosis ($h_{jj'}$)	21	0.02	9.76***	572.29*	21	10.71***	0.01	21	0.03	310.54	6.58*	21	12.69***	541.25*	0.02
Average heterosis ($\bar{h}$)	1	0.08*	114.80***	2863.30*	1	15.71	0.00	1	0.07	1656.02	37.33*	1	169.64***	4718.72**	0.20**
Variety heterosis (h_j)	6	0.02	3.24	925.03**	6	6.62***	0.02	6	0.01	476.89	0.53	6	3.17	720.72*	0.03
Specific heterosis ($s_{jj'}$)	14	0.01	5.04*	257.48	14	12.49***	0.01	14	0.03	143.14	6.98	14	5.60*	165.94	0.01
E × Entries	243	0.02	2.52	367.06*	54	6.36	0.02**	81	0.02	246.74	3.78	540	3.33	371.98***	0.03
E × v_j	54	0.02	3.66	524.81**	12	12.11*	0.04**	18	0.04	381.88	4.62	120	4.62*	510.74***	0.03
E × $h_{jj'}$	189	0.02	2.19	321.99	42	0.99	0.02	63	0.02	208.12	3.54	420	2.91	332.33**	0.03
E × $\bar{h}$	9	0.01	1.71	424.91	2	14.74	0.00	3	0.03	713.75*	0.51	20	3.09	428.26	0.02
E × h_j	54	0.02	1.94	253.67	12	0.14	0.02	18	0.03	175.63	3.89	420	2.14	307.65	0.03
E × $s_{jj'}$	126	0.02	2.34	343.92	28	0.18	0.02*	42	0.02	185.94	3.61	280	3.23	336.06**	0.03
Pooled error	486	0.02	2.71	299.69	101	5.23	0.01	216	0.02	236.19	4.16	1017	3.39	255.39	0.03

*,**,*** Significant at $P < 0.05$, $P < 0.01$, and $P < 0.001$, respectively.

[†]df for AD were 8, 2, and 16 under optimal, managed low N, and across environments, respectively.

[†]df for EPP were 1 and 19, under managed drought and across environments, respectively.

[†]df for PH were 7 and 12, under optimal and across environments, respectively.

[‡]AD, days to anthesis; EPP, ears per plant; and PH, plant height.

Supplemental Table S3. Estimates of variety effects (v_j), variety heterosis (h_j), general combining ability (GCA) effects, and variety means for days to anthesis and plant height under different management options and across environments over two years.

(a) Days to anthesis

	Optimal conditions				Managed drought				Managed low N				Across environments			
Population	v_j	h_j	GCA	Mean	v_j	h_j	GCA	Mean	v_j	h_j	GCA	Mean	v_j	h_j	GCA	Mean
	d				d				d				d			
ECAVL1	0.51	-0.38	-0.12	78	0.61	-0.57	-0.27	85	-0.22	-0.09	-0.20	84	0.30	-0.23	-0.09	79
ECAVL2	0.71	0.16	0.52	78	0.30	0.14	0.30	86	1.67	-0.03	0.80	86	0.86	0.09	0.51	80
ECAVL16	-1.89	0.24	-0.70	76	0.25	-0.80	-0.68	83	-1.44	0.03	-0.69	82	-1.65	0.13	-0.66	77
ECAVL16-STR	1.71	-0.28	0.58	79	0.88	-0.42	0.02	86	0.78	0.14	0.53	84	1.34	-0.22	0.49	80
ECAVL17	0.35	0.09	0.27	78	-1.84	1.10	0.18	84	0.44	0.27	0.49	85	0.11	0.22	0.29	79
ECAVL18	0.27	0.19	0.32	78	0.02	0.71	0.72	86	0.67	-0.11	0.22	85	0.58	-0.01	0.28	80
NIP25	-1.69	-0.02	-0.86	76	-0.22	-0.15	-0.26	84	-1.88	-0.21	-1.16	82	-1.54	0.02	-0.82	78
SE/LSD $_{(0.05)}$ †	1.26	0.87	0.56	1	7.64	5.10	3.34	3	1.62	1.08	0.72	2	1.34	0.94	0.61	1
Average heterosis		1.01***				-0.69				-0.89				-0.84		

(b) Plant height

Population	Optimal conditions				Managed low N				Across environments			
	v_j	h_j	GCA	Mean	v_j	h_j	GCA	Mean	v_j	h_j	GCA	Mean
	cm				cm				cm			
ECAVL1	0.05	3.22	3.24	200	9.15	-5.44	-0.87	192	2.05	0.05	1.08	198
ECAVL2	10.22	-6.10	-0.98	208	14.32	-3.54	3.61	195	10.32	-3.98	1.17	205
ECAVL16	-2.08	3.37	2.33	196	5.23	2.83	-0.20	187	2.05	-0.31	0.72	197
ECAVL16-STR	5.44	-3.00	-0.28	204	2.15	0.34	1.41	185	4.37	-1.27	0.92	200
ECAVL17	-10.21	5.55	0.44	189	-13.51	6.74	-0.02	169	-11.47	5.78	0.05	184
ECAVL18	3.09	0.11	1.66	200	4.82	2.01	4.41	187	3.87	0.08	2.01	199
NIP25	-6.51	-3.15	-6.41	191	-22.15	2.74	-8.35	161	-11.18	-0.35	-5.94*	184
SE/LSD $_{(0.05)}$ †	8.63	5.79	3.87	11	9.57	6.42	4.28	13	6.22	4.17	2.78	9
Average heterosis	4.87				5.12				4.86			

(c) Ears per plants

Population	Optimal conditions				Managed drought				Managed low N				Across environments			
	v_j	h_j	GCA	Mean	v_j	h_j	GCA	Mean	v_j	h_j	GCA	Mean	v_j	h_j	GCA	Mean
		no				no				no				no		
ECAVL1	-0.02	0.00	-0.01	1.0	-0.03	0.03	0.02	0.2	-0.0	-0.02	-0.01	0.9	-0.01	-0.01	-0.01	0.9
ECAVL2	0.06	-0.01	0.02	1.1	0.12	-0.06	0.00	0.4	-0.01	0.02	0.02	0.9	0.03	0.00	0.02	0.9
ECAVL16	-0.03	0.00	-0.02	1.0	-0.02	-0.04	-0.05	0.2	-0.02	0.01	0.00	0.9	-0.02	-0.01	-0.02	0.9
ECAVL16-STR	0.06	-0.03	0.00	1.1	-0.05	0.05	0.02	0.2	0.02	-0.04	-0.02	1.0	0.03	-0.02	0.00	0.9
ECAVL17	-0.10**	0.00	-0.05**	1.0	-0.07	0.02	-0.02	0.2	-0.04	0.03	0.01	0.9	-0.06	-0.01	-0.04*	0.8
ECAVL18	0.08*	0.01	0.05***	1.1	0.07	-0.02	0.02	0.3	0.09*	-0.01	0.03	1.0	0.05	-0.01	0.04*	1.0
NIP25	-0.06	0.03	0.00	1.0	-0.01	0.02	0.01	0.2	-0.04	-0.04	-0.02	0.9	-0.04	0.01	0.01	0.9
SE/LSD $_{(0.05)}$ †	0.04	0.02	0.02	0.1	0.07	0.05	0.03	0.2	0.05	0.04	0.02	0.1	0.04	0.02	0.02	0.1
Average heterosis		0.02				0.01				0.03				0.03		

*,**,*** Significant at $P < 0.05$, $P < 0.01$, and $P < 0.001$, respectively.

†SE standard error of variety, heterosis and GCA effects; LSD for mean grain yield.

Supplemental Table S4. Mean days to anthesis, plant height and ear per plant of 21 population hybrids evaluated under optimal, managed low N, and managed drought stress for two years (2008 and 2009).

		Optimal			Managed low N			Managed drought	
Entry	Pedigree	AD	PH	EPP	AD	PH	EPP	AD	EPP
1	ECAVL1 × ECAVL2	78	203	1.1	84	199	1.1	86	0.3
2	ECAVL1 × ECAVL16	76	211	1.0	81	198	1.0	82	0.2
3	ECAVL1 × ECAVL16-STR	77	204	1.1	82	196	0.9	85	0.3
4	ECAVL1 × ECAVL17	78	206	1.0	84	194	1.0	84	0.3
5	ECAVL1 × ECAVL18	76	206	1.1	81	194	0.9	84	0.3
6	ECAVL1 × NIP25	76	204	1.0	81	190	1.0	84	0.2
7	ECAVL2 × ECAVL16	77	206	1.1	83	200	1.0	84	0.2
8	ECAVL2 × ECAVL16-STR	78	200	1.1	83	198	1.0	86	0.3
9	ECAVL2 × ECAVL17	78	203	1.1	84	196	0.9	85	0.2
10	ECAVL2 × ECAVL18	78	209	1.1	82	202	1.1	85	0.3
11	ECAVL2 × NIP25	76	196	1.1	80	195	0.9	84	0.2
12	ECAVL16 × ECAVL16-STR	77	201	1.1	82	195	1.0	83	0.3
13	ECAVL16 × ECAVL17	76	209	1.0	82	192	1.0	83	0.3
14	ECAVL16 × ECAVL18	77	203	1.1	82	200	1.0	83	0.2
15	ECAVL16 × NIP25	76	198	1.1	81	189	1.0	83	0.3
16	ECAVL16-STR × ECAVL17	77	208	1.0	83	202	1.0	85	0.2
17	ECAVL16-STR × ECAVL18	78	206	1.1	83	201	0.9	84	0.3
18	ECAVL16-STR × NIP25	76	200	1.1	83	186	0.9	84	0.4
19	ECAVL17 × ECAVL18	77	202	1.1	82	199	1.1	85	0.3
20	ECAVL17 × NIP25	76	194	1.0	83	186	0.9	84	0.2
21	ECAVL18 × NIP25	77	197	1.1	82	190	1.0	84	0.3
	LSD (0.05)	1	10	0.1	2	16	0.1	2	0.2
	Heritability	0.92	0.52	0.75	0.62	0.40	0.23	0.41	0

Supplemental Table S5. Pearson correlation coefficients between different heterosis measures†, and between heterosis and genetic distance under different environments.

	MPHOPT	HPHOPT	MPHLN	HPHLN	MPHMDR	HPHMDR	MPHACR	HPHACR
HPHOPT	0.87***							
MPHLN	0.37	0.19						
HPHLN	0.31	0.41	0.59					
MPHMDR	0.27	0.38	0.36	0.62**				
HPHMDR	0.19	0.38	0.24	0.48	0.93***			
Genetic distance	0.25	0.16	0.40	0.14	0.21	0.15	0.28	0.12

,* Significant at $P < 0.01$, and $P < 0.001$, respectively.

†HPHACR, high parent heterosis across environments; HPHLN, high parent heterosis under low N; HPHMDR, high parent heterosis under managed drought; HPHOPT, high parent heterosis under optimal environments; MPHACR, mid-parent heterosis across environments; MPHLN, mid-parent heterosis under low N; MPHMDR, mid-parent heterosis under managed drought; MPHOPT, mid-parent heterosis under optimal environments.
